# Impulsivity, Psychological Distress, and Quality of Life Among the Patients With Acne Vulgaris: Factors Related to Acne Scars

**DOI:** 10.1111/jocd.70838

**Published:** 2026-04-02

**Authors:** Halil İbrahim Yanik, Ersoy Acer, İmran Gökçen Yilmaz Karaman, Esra Ağaoğlu, Hilal Kaya Erdoğan, Muzaffer Bilgin, Zeynep Nurhan Saraçoğlu

**Affiliations:** ^1^ Dermatology and Venereology Department Muş State Hospital Muş Turkey; ^2^ Dermatology and Venereology Department Eskisehir Osmangazi University Eskisehir Turkey; ^3^ Psychiatry Department Eskisehir Osmangazi University Eskisehir Turkey; ^4^ Biostatistics Department Eskisehir Osmangazi University Eskisehir Turkey

**Keywords:** acne vulgaris, cosmetics, impulsivity, quality of life, risk factors

## Abstract

**Background:**

Acne scars affect up to 95% of acne vulgaris (AV) patients. However, there is little clinical data to predict which patients are more prone to create scars.

**Objective:**

In this study, we aimed to evaluate the impulsivity, psychological distress, and quality of life in acne patients and factors related to acne scars.

**Methods:**

This study included 403 AV patients aged 18 years and older. The Global Evaluation Acne (GEA) Scale and the Global Scale for Acne Scar Severity (SCAR‐S) were used to assess acne and scar severity. The patients completed a form that included epidemiologic data, such as smoking habits, alcohol consumption, acne characteristics, the Acne Quality of Life Instrument (AQLI), the Barratt Impulsivity Scale‐11 Short Form (BIS‐11‐SF), and the Depression Anxiety Stress Scale‐21 (DASS‐21).

**Results:**

Of the 403 acne patients, 56% (*n* = 226) had scars. The male sex, mean disease duration, and severity of acne were higher, and the age of acne onset was lower in the group with acne scars (*p* < 0.001, *p* = 0.030, *p* < 0.001, *p* < 0.001). Oily skin type was more frequent in patients with acne scars (*p* < 0.001), and Fitzpatrick skin types 3 and 4 were more frequent in patients with acne scars (*p* < 0.001, *p* < 0.001). Pruritus and postinflammatory hyperpigmentation were more frequent in the group with acne scars (*p* < 0.001, *p* < 0.001). The time from the first acne lesion to the beginning of treatment was longer in the group with acne scars (*p* < 0.001). The history of systemic drug, topical drug, and dermo‐cosmetic product use for acne treatment was lower in the group with acne scars (*p* = 0.010, *p* < 0.001, *p* < 0.001, *p* < 0.001). The mean AQLI, BIS‐11‐SF, total DASS‐21, depression subscale, and stress subscale scores were higher in the group with acne scars (*p* < 0.001, *p* < 0.001, *p* = 0.002, *p* = 0.007, *p* = 0.002). There was a negative correlation between scar severity score and the age of acne onset and a positive correlation between scar severity score and disease duration (*p* < 0.001, *r* = 0.16, *p* < 0.001, r = −0.3, respectively). There was a positive correlation between scar severity score and the mean AQLI, BIS‐11‐SF, total DASS‐21, depression subscale, and stress subscale (*p* < 0.001, *r* = 0.2, *p* < 0.001, *r* = 0.16, *p* < 0.001, *r* = 0.16; *p* < 0.001, *r* = 0.17, *p* = 0.003, *r* = 0.15, respectively).

**Conclusion:**

Male sex, early onset of acne, prolonged acne duration, severe acne, delayed treatment, oily skin type, darker skin type, presence of pruritus, and higher impulsivity were associated with acne scars. The presence of scars had adverse effects on quality of life and psychoemotional status, such as depression and stress.

## 1 Introduction

Acne is a chronic disease of the pilosebaceous unit in which non‐inflammatory and inflammatory lesions such as comedones, papules, pustules, and nodules occur. It typically begins to be noticeable in adolescence and may persist into adulthood. It has an estimated prevalence of 9.38% worldwide [1, 2, 3]. Four main topics are involved in the pathogenesis of acne: abnormal follicular keratinization, excessive sebum production, inflammation, and colonization by *Propionibacterium acnes* [4]. As a result of follicular rupture, abnormal wound healing may occur during the inflammatory process, and acne scars affect up to 95% of individuals with acne. All types of acne can leave scars. Acne scars disrupt the cosmetic appearance, decrease patients' quality of life, and affect their psychosocial development [5, 6].

Completely treating acne scars can be quite challenging. Therefore, it is essential to prevent scars from occurring. It is crucial to know patients who are prone to scars. However, very few studies evaluate the factors associated with acne scars. Genetic factors, acne severity and duration, degree of inflammation of the lesions, delay in treatment, the habit of squeezing and picking the lesions, and male sex have been associated with the development of acne scars. There are conflicting results regarding the effect of diet on the formation of acne scars. In addition, there is very little data on the relationship between the presence of acne scars and impulsivity, depression, anxiety, and stress [5, 7, 8]. In this study, we aimed to evaluate the epidemiological factors associated with acne scars and the psychological and emotional status of patients with acne scars.

## 2 Materials and Methods

The study employed a case–control methodology conducted cross‐sectionally. All participants gave informed consent prior to the present study.

A total of 403 patients with acne, aged 18 years and older, were included in the study. Patients with any psychiatric disease, eating disorder, remarkable diet history, chronic diseases such as hypertension, renal insufficiency, thyroid disease, obesity, and cancer, those who were cognitively incapable of completing the questionnaires alone, under 18 or over 65 years of age, and pregnant or breastfeeding mothers were not included in the study.

A dermatologist evaluated the Fitzpatrick skin type, acne severity, scar presence, and scar severity through a dermatologic examination using the Global Evaluation Acne (GEA) Scale and the Global Scale for Acne Scar Severity (SCAR‐S).

The GEA Scale was developed by Dreno et al. in 2011 and updated in 2016. Acne lesions are evaluated as 0 “No lesion,” 1 “Almost no lesion,” 2 “Mild,” 3 “Moderate,” 4 “Severe,” and 5 “Very severe” [9, 10].

SCAR‐S is a practical system for evaluating acne scars on the face, chest, and back. In the chart developed by Tan et al., scars on the face, back, and chest are scored between 0 and 5 according to clinical appearance and prevalence. The scoring for each region is summed, and a general score between 0 and 15 is obtained [11].

The patients completed a form that included sociodemographic data, such as education level, smoking habits, and alcohol consumption, as well as the Acne Quality of Life Instrument (AQLI), the Barratt Impulsivity Scale‐11 Short Form (BIS‐11‐SF), and the Depression Anxiety Stress Scale‐21 (DASS‐21).

BIS‐11‐SF consists of 15 questions. The scale has three sub‐dimensions, including attentional impulsivity (5 items), motor impulsivity (5 items), and lack of planning (5 items) [12]. A Turkish validity and reliability study was conducted by Güleç et al. in 2008 [13].

The total scores of the quality of life scale are determined by summing the scores assigned to each question in the AQLI. Tuncayengin et al. conducted a validity and reliability study in Turkish in 2010 [14].

The DASS‐21 scale has seven questions each to measure the dimensions of depression, stress, and anxiety. It is a 4‐point Likert‐type scale coded as 0 “not suitable for me,” 1 “somewhat suitable for me,” 2 “usually suitable for me,” and 3 “completely suitable for me.” The Turkish validity and reliability study was conducted by Yılmaz et al. in 2017 [15, 16].

### 2.1 Statistical Analysis

Continuous data are given as Mean ± Standard Deviation. Categorical data are presented as percentages (%). The Shapiro–Wilk test was used to investigate the suitability of the data for a normal distribution. An independent sample *t*‐test analysis was used to compare two normally distributed groups. When comparing groups that do not conform to a normal distribution, the Mann–Whitney *U* test is used for cases involving two groups. Spearman correlation coefficients were calculated for variables that did not conform to normal distribution to determine the direction and magnitude of the relationship (correlation) between variables. Pearson's chi‐squared analysis was used to analyze the created cross‐tables. IBM SPSS Statistics 21.0 (IBM Corp. Released 2012. IBM SPSS Statistics for Windows, Version 21.0. Armonk, NY: IBM Corp.) program was used. A value of *p* < 0.05 was accepted as the criterion for statistical significance.

## 3 Results

### 3.1 Sociodemographic Characteristics of Patients According to the Presence of Acne Scar

Of 403 acne patients included in the study, 56% (*n* = 226) had acne scars, and 44% (*n* = 177) did not. Of the 226 patients with acne scars, 31.9% (*n* = 72) were male and 68.1% (*n* = 154) were female. Of the 177 patients without acne scars, 13.6% (*n* = 24) were male and 86.4% (*n* = 153) were female. The male patients were more prevalent in the group with acne scars (*p* < 0.001). The mean age was 22.3 ± 4.45 years in the group with acne scars and 23.28 ± 5.33 years in the group without acne scars (*p* = 0.05).

The mean acne duration was longer in the group with acne scars (6.78 ± 4.61 years) than in the group without acne scars (6.4 ± 5.55 years) (*p* = 0.03) (Table 1). The age of acne onset was lower in the group with acne scars (15.53 ± 2.72 years) than in the group without acne scars (16.89 ± 3.75 years) (*p* < 0.001) (Table 1).

**TABLE 1:** Sociodemographic and clinical characteristics of patients according to the presence of scar.

		*n* (%), mean ± standard deviation, median (p25–p75)		*p*
		Presence of scar	Absence of scar	
*Characteristics*				
Sex	Female	154 (68.1%)	153 (86.4%)	**< 0.001** ^a^
	Male	72 (31.9%)	24 (13.6%)	
Age		22.3 ± 4.45 21 (19–23.75)	23.28 ± 5.33 22 (20–25)	0.05^b^
BMI		22.47 ± 4.21 21.8 (19.9–23.5)	21.89 ± 2.98 21.6 (19.8–23.5)	0.38^b^
Disease duration (years)		6.78 ± 4.61 6 (4–9)	6.4 ± 5.55 5 (3–9)	**0.03** ^c^
Age of disease onset (years)		15.53 ± 2.72 15 (14–16)	16.89 ± 3.75 16 (15–18)	**< 0.001** ^c^
Marital status	Married	16 (7.1%)	21 (11.9%)	0.14^a^
	Single	210 (93.9%)	156 (88.1%)	
Education level	Primary School	1 (0.4%)	1 (0.6%)	0.84^a^
	Middle School	1 (0.4%)	1 (0.6%)	
	High School	37 (16.4%)	27 (15.2%)	
	University	174 (77%)	142 (80.2%)	
	Graduate	13 (5.8%)	6 (3.4%)	
Smoking status	Smoking	55 (24.3%)	28 (15.8%)	0.05^a^
	Non‐smoking	171 (75.7%)	149 (84.2%)	
Alcohol consumption	Yes	69 (30.5%)	49 (27.7%)	0.61^a^
	No	157 (69.5%)	128 (72.3%)	
Skin type	Dry	43 (19%)	33 (18.6%)	**< 0.001** ^a^
	Combination	98 (43.4%)	104 (58.8%)	
	Oily	85 (37.6%)	40 (22.6%)	
Family history of severe acne	Yes	115 (50.9%)	75 (42.4%)	0.11^a^
	No	111 (49.1%)	102 (57.6%)	
Fitzpatrick skin type	1	25 (11.1%)	22 (12.4%)	**< 0.001** ^a^
	2	87 (38.5%)	101 (57.1%)	
	3	84 (37.2%)	43 (24.3%)	
	4	30 (13.3%)	11 (6.2%)	
History of pre‐adolescent acne	Yes	24 (10.6%)	10 (5.6%)	0.11^a^
	No	202 (89.4%)	167 (94.4%)	
Time to treatment initiation	0–6 months	43 (19%)	84 (47.5%)	**< 0.001** ^a^
	6‐12 months	20 (8.9%)	40 (22.6%)	
	1–3 years	52 (23%)	44 (24.9%)	
	3–10 years	97 (42.9%)	9 (5.1%)	
	More than 10 years	14 (6.2%)	0 (0%)	
GEA Scale	0	1 (0.4%)	11 (6.2%)	< 0.001^a^
	1	16 (7.1%)	51 (28.8%)	
	2	45 (19.9%)	70 (39.6%)	
	3	99 (43.8%)	43 (24.3%)	
	4	62 (27.4%)	2 (1.1%)	
	5	3 (1.3%)	0 (0%)	
Itching	Yes	118 (52.2%)	27 (15.3%)	**< 0.001** ^a^
	No	108 (47.8%)	150 (84.7%)	
Post‐inflammatory hyperpigmentation	Yes	195 (86.3%)	69 (39%)	**< 0.001** ^a^
	No	31 (13.7%)	108 (61%)	
History of systemic medication use	Yes	102 (45.1%)	103 (58.2%)	**< 0.01** ^a^
	No	124 (54.9%)	74 (41.8%)	
History of topical treatment use	Yes	164 (72.6%)	160 (90.4%)	**< 0.001** ^a^
	No	62 (27.4%)	17 (9.6%)	
Use of dermocosmetic products	Yes	167 (73.9%)	159 (89.8%)	**< 0.001** ^a^
	No	59 (26.1%)	18 (10.2%)	
History of cosmetic procedures	Yes	29 (12.8%)	26 (14.7%)	0.69^a^
	No	197 (87.2%)	151 (85.3%)	

Abbreviations: BMI, body mass index; GEA scale, global evaluation acne scale. A Bold value of *p*〈 0.05 was accepted as the criterion for statistical significance.

^a^ Chi‐square test.

^b^ *t*‐test.

^c^ Mann–Whitney *U* test.

Of the 226 patients with acne scars, 93.4% (*n* = 211) had ice pick scars, 76.6% (*n* = 173) had box scars, 69.5% (*n* = 157) had rolling scars, and 11.1% (*n* = 25) had hypertrophic and keloid scars. 97% (*n* = 219) of the patients had scars on the cheek, 81% (*n* = 183) on the forehead, 64.6% (*n* = 146) on the back, 46.5% (*n* = 105) on the chest, 34.5% (*n* = 78) on the submental region, 31.4% (*n* = 71) on the submandibular region, and 27.9% (*n* = 63) on the nose.

Oily skin type was more frequent in patients with acne scars (36.7%) than in patients without scars (22.6%) (*p* < 0.001). In addition, Fitzpatrick skin types 3 and 4 were more common in patients with acne scars compared to those without scars (*p* < 0.001) (Table 1).

When we evaluated the acne severity of the patients according to the GEA Scale, 43.8% (*n* = 99) of the patients had a severity score of 3, and 27.4% (*n* = 62) had a severity score of 4 in patients with acne scars; 24.3% (*n* = 43) of the patients had a severity score of 3% and 1.1% (*n* = 2) had a severity score of 4 in patients without acne scars. The severity of acne was higher in patients with acne scars than in patients without acne scars (*p* < 0.001) (Table 1).

Pruritus was present in 52.2% (*n* = 118) of patients with acne scars and 15.3% (*n* = 27) of patients without acne scars. In addition, 86.3% (*n* = 195) of patients with acne scars and 39% (*n* = 69) of patients without acne scars experienced postinflammatory hyperpigmentation. The presence of pruritus and postinflammatory hyperpigmentation was higher in the group with acne scars than in patients without acne scars (*p* < 0.001, *p* < 0.001) (Table 1).

The time from the first acne lesion to the beginning of treatment was longer in the group with acne scars (*p* < 0.001). The history of systemic drug usage for acne treatment was lower in the group with acne scars (45.1%) compared to the group without acne scars (58.2%), with a statistically significant difference (*p* = 0.010). The history of topical medication usage for acne treatment was lower in the group with acne scars (72.6%) compared to the group without acne scars (90.4%) (*p* < 0.001). The history of dermo‐cosmetic product usage was lower in the group with acne scars (73.9%) compared to the group without acne scars (89.8%) (*p* < 0.001). See Table 1 for an overview.

### 3.2 Impulsivity, Psychological Distress Levels, and Quality of Life According to the Presence of Scar

The mean AQOLS score was higher in the group with acne scars (18.12 ± 9.15) than in the group without acne scars (14.68 ± 9.71) (*p* < 0.001). The mean BIS‐11 score was higher in the group with acne scars (28.42 ± 7.26) than in the group without acne scars (25.86 ± 7.01) (*p* < 0.001). The mean depression subscale, stress subscale, and total DASS‐21 scores were higher in the group with acne scars (5.49 ± 4.34), (7.68 ± 4.75), (18.15 ± 11.28) than in the group without acne scars (4.33 ± 4.16), (6.38 ± 4.29), (14.86 ± 10.08) (*p* = 0.002, *p* = 0.007, *p* = 0.002, respectively). The mean anxiety subscale score was also higher in the group with acne scars (5.02 ± 3.96) than in the group without scars (4.2 ± 3.19), but this difference was not statistically significant (*p* = 0.090). Table 2 summarizes the differences of impulsivity, psychological distress levels, and quality of life.

**TABLE 2:** Impulsivity, psychological distress levels, and quality of life according to the presence of scar.

	Mean ± standard deviation, median (p25–p75)		*p*
	Presence of scar	Absence of scar	
Total DASS‐21	18.15 ± 11.28 17 (10–24)	14.86 ± 10.08 13 (7–22)	**0.002** ^a^
Depression subscale	5.49 ± 4.34 5 (2–8)	4.33 ± 4.16 3 (1–6)	**0.002** ^a^
Anxiety subscale	5.02 ± 3.96 4 (2–7)	4.2 ± 3.19 4 (2–6)	0.09^a^
Stress subscale	7.68 ± 4.75 7 (4–10.8)	6.38 ± 4.29 6 (3–9)	**0.007** ^a^
AQLI	18.12 ± 9.15 18 (11–24.75)	14.68 ± 9.71 13 (7–22)	**< 0.001** ^a^
BIS‐11‐SF	28.42 ± 7.26 27 (24–32)	25.86 ± 7.01 25 (21–31)	**< 0.001** ^a^

Abbreviations: AQLI, acne quality of life instrument; BIS‐11‐SF, barratt impulsivity scale‐11 short form; DASS‐21, depression anxiety stress scale‐21. A Bold value of *p*〈 0.05 was accepted as the criterion for statistical significance.

^a^ Mann–Whitney *U* test.

### 3.3 Correlation of SCAR‐S and the Other Parametric Data

We found an inverse relationship between SCAR‐S and the age of acne onset, as well as a direct relationship between SCAR‐S and acne duration (*p* < 0.001, r = −0.300; *p* < 0.001, *r* = 0.160, respectively). There was a direct relationship between SCAR‐S and AQLI, BIS‐11, total DASS‐21, depression subscale, and stress subscale scores (*p* < 0.001, *r* = 0.200; *p* < 0.001, *r* = 0.160; *p* < 0.001, *r* = 0.160; *p* < 0.001, *r* = 0.170; *p* = 0.003, *r* = 0.150, respectively). Table 3 demonstrates correlation analyses.

**TABLE 3:** Correlation of GEA Scale and the other parametric data.

	*r*; *p* ^a^
	GEA scale
Age	−0.08; 0.09
Age of disease onset	**−0.3; < 0.001**
Disease duration	**0.16; < 0.001**
BMI	0.08; 0.11
AQLI	**0.2; < 0.001**
BIS‐11‐SF	**0.16; < 0.001**
Total DASS‐21	**0.16; < 0.001**
Depression subscale	**0.17; < 0.001**
Anxiety subscale	0.08; 0.12
Stress subscale	**0.15; 0.003**

Abbreviations: AQLI, acne quality of life instrument; BIS‐11‐SF, barratt impulsivity scale‐11 short form; BMI, body mass index; DASS‐21, depression anxiety stress scale‐21; GEA Scale, global evaluation acne scale. A Bold value of *p*〈 0.05 was accepted as the criterion for statistical significance.

^a^ Spearman correlation.

## 4 Discussion

The present study found that 56% of the participants with AV had acne scars. The development of acne scars was related to male sex, early age of acne onset, prolonged acne duration, severe acne, delayed treatment, oily skin type, darker skin type, and presence of pruritus. Regarding psychodermatological evaluation, patients with acne scars had higher psychological distress, lower quality of life, and higher impulsivity levels.

The pathogenesis of scar development in acne is not fully understood, although it is associated with follicle rupture, abnormal wound healing, and inflammation [17]. Scars do not develop in every patient with acne, and there is not always a correlation with the clinical severity of the disease. There is limited clinical data to predict which acne patients are more likely to experience scarring [18]. It may be crucial to identify patients who are prone to scarring so we can prevent the occurrence of acne scars. The literature reports that acne scars are more common in male patients [7]. One study found acne scars in 58% of males and 46% of females [19]. In our study, the proportion of male patients was also higher in the group with acne scars. Patients with severe acne often have a positive family history of severe acne. A study reported that acne and acne scars were more prevalent in individuals with a family history of severe acne [20]. However, in our study, no significant relationship was found between a family history of severe acne and the presence of scars.

Studies have reported a positive correlation between high sebum levels, oily skin type, and the severity of acne scars [21, 22]. People with oily skin type are 4.3 times more likely to develop acne scars than those with dry skin [22]. Our study also showed that oily skin type was more frequent in patients with acne scars. The prevalence of inflammatory acne increases as the skin phototype darkens. Furthermore, acne patients with darker skin types are more likely to experience post‐inflammatory hyperpigmentation and keloidal or hypertrophic scars [23]. A study by Perkins et al. reported a higher prevalence of acne sequelae, such as acne scars and dyspigmentation, in patients with darker skin tones [24]. In our study, Fitzpatrick skin types 3 and 4 were more frequent in patients with acne scars, while Fitzpatrick skin type 2 was more common in patients without acne scars.

Acne severity and scars are associated with disease duration, age of onset, and delay in treatment. Tan et al. found that acne severity was the most crucial factor in acne scar development [25]. In our research, acne severity was also higher in the group with acne scars compared to the group without scars. According to the GEA scale, acne scars were statistically significantly higher, particularly in patients with a severity score of 3 or higher.

As the duration of acne increases, the likelihood of developing acne scars also increases. A study grouped patients into two categories: those with acne duration longer than 1 year and those with acne duration less than 1 year. They found that acne scars were higher in the group with acne duration longer than 1 year [25]. Layton et al. demonstrated that untreated acne disease, lasting up to 3 years, is crucial in determining scar formation. After this time interval, the degree of scars increases significantly [5]. In our study, the duration of acne was longer in patients with acne scars, and the age of onset of acne was lower. Moreover, the scar severity score was negatively correlated with the age of onset of acne and positively correlated with the duration of the acne. Dessinioti et al. found that the onset age of acne was earlier in patients with acne scars; however, this difference was not statistically significant [7]. Another study by Hayashi et al. found that the onset age of acne was similar in both groups with and without scars. Further, they found that the duration of acne was longer in the group with acne scars [26].

Another problem associated with acne is hyperpigmentation, which often occurs after acne lesions have healed. Female sex, darker skin type, severe acne, facial areas, excessive exposure to sunlight, and squeezing or scratching lesions are important risk factors for post‐acne hyperpigmentation. Pruritus is a common symptom in patients with acne, and scar formation is more prevalent in lesions that have been traumatized by scratching [27]. Al‐Qarqaz et al. demonstrated that the incidence of post‐acne hyperpigmentation increased in proportion to the severity of acne, and a significant relationship was found between pruritus and post‐acne hyperpigmentation [27]. In our study, pruritus and post‐inflammatory hyperpigmentation were more frequent in patients with acne scars. Therefore, taking preventive measures regarding pruritus in acne patients may be substantial.

Acne scars are primarily observed in moderate to severe inflammatory acne lesions; however, scar development can also be seen in some mild, non‐inflammatory lesions. A connection exists between early acne management and the prevention of scarring [28]. An immunohistochemical case–control study has demonstrated that the duration of the inflammatory phase is prolonged, and the specific immune response is enhanced in patients with scars. This study suggested that early treatment of inflammatory acne lesions could prevent acne scars [29]. In our study, another factor associated with scar formation was the delay in treatment. Acne scars were more frequent in patients treated more than 3 years after the onset of acne. Therefore, patients with acne should be treated early to prevent scar development. In our study, a history of prior systemic treatment (including doxycycline, azithromycin, tetracycline, systemic isotretinoin, and combined oral contraceptives) was more common in the group without acne scars. Additionally, acne scars were lower in patients with a history of doxycycline usage. This suggests that systemic treatment, especially doxycycline, may protect against the development of acne scars due to its anti‐inflammatory effect.

In our study, the history of topical treatment usage was higher in patients without acne scars. It may be related to the more frequent use of topical agents in patients with mild acne and the lower incidence of scar formation in patients with mild acne. Moreover, a history of topical treatment can serve as a protective factor for acne scars. Dermo‐cosmetics have become increasingly critical in dermatology and are emerging as an alternative treatment strategy for patients with long‐term illnesses and during relapse periods [30]. A study by Lucas et al. found that adherence to dermo‐cosmetic treatment resulted in a 2.2‐fold increase in the likelihood of adherence to topical pharmacological treatment and was associated with a significant reduction in the severity and number of acne lesions [31]. In our study, we questioned patients about their use of dermo‐cosmetic products (face wash gel, exfoliating products, moisturizer, sunscreen); dermo‐cosmetic product usage was higher in the group without acne scars. Adherence to dermo‐cosmetic treatment may also increase adherence to acne treatment, thereby helping to prevent scar development.

Acne scars are permanent and can have a negative impact on a patient's appearance, quality of life, and psychosocial development. Impulsivity is a multifaceted and complex concept involving behavioral, cognitive, and neurophysiological components. The definition of impulsivity is “behaviors that are inadequately planned, prematurely expressed, excessively risky or inappropriate to the situation, and that often result in undesirable consequences” [32]. It can lead to skin‐picking [33]. Acne is one of the most commonly known triggers of skin‐picking disorder. Excoriation of acneiform lesions also results in hyperpigmentation, scars, and more acne [34]. One study found that impulsivity increased as acne severity increased, but this result was not statistically significant [35]. To our knowledge, no study in the literature has evaluated acne scars and impulsivity. In our research, the impulsivity score was higher in the group with acne scars, and a positive correlation was also found between acne scar severity and impulsivity. Acne treatments themselves might influence impulsivity levels. There is limited but relevant literature suggesting that systemic treatments such as isotretinoin do not significantly alter impulsivity in patients with moderate‐to‐severe acne vulgaris [36]. So that the increased impulsivity in our study is more likely linked to the presence of scars and their psychosocial impact rather than pharmacological treatment effects. Identifying acne patients with high impulsivity and providing psychiatric support for it may help prevent scar formation.

Acne negatively impacts the quality of life. Moreover, as the severity of acne scars increases, their quality of life is adversely affected [37]. In our study, the quality of life was poorer in the group with acne scars, and a positive correlation was found between scar severity and quality of life. Acne often affects young people at a time when they are experiencing maximum psychological, social, and physical changes. The face is frequently affected, and since facial appearance represents an essential aspect of an individual's body image perception, a sensitive individual with facial acne may develop significant psychosocial problems due to the disease process. Emotional stress can exacerbate acne, and psychiatric issues can develop in patients with acne [38]. A study by Yazıcı et al. found that depression and anxiety were higher in patients with acne [39]. Furthermore, a study presented a positive correlation between acne severity and anxiety and depression [40]. In a survey by Yolaç Yarpuz et al., social anxiety, social avoidance, general anxiety, depression, and faulty automatic thoughts were higher, and self‐esteem was lower in patients with acne [41]. Acne scars, which are permanent sequelae, further negatively affect the patients' psychoemotional status. In our study, the total DASS‐21 score, depression, and stress subscales were higher in the patients with scars. In addition, there was a positive correlation between acne scar severity and the total DASS‐21 score, stress, and depression subscales. In patients with acne, the presence of scars and increasing scar severity have a greater negative impact on quality of life, and psychoemotional disorders such as depression and stress are more frequent. Therefore, it is crucial to evaluate the psychoemotional status, especially in acne patients with scars.

Limitations of our study were the cross‐sectional design, which prevents establishing causal relationships; the use of only self‐report measures for impulsivity assessment; inclusion of patients regardless of their ongoing treatments; and the data being collected from a single center, which may limit generalizability.

In conclusion, the development of acne scars is not rare. Since acne scars are a preventable medical condition, clinicians should aim to treat AV in its early phases. Acne scars have adverse effects on psychological status; effective treatments of AV and prevention of acne scars can also result in better mental health. Among the patients with AV, those with impulsive traits may be at risk for acne scar development.

## Author Contributions

Wholly authors read and accepted the last manuscript. H.İ.Y., E.A., İ.G.Y.K., E.Ağ., H.K.E., M.B., and Z.N.S. designed the research study, H.İ.Y., E.A., İ.G.Y.K., E.Ağ., H.K.E., M.B., and Z.N.S. performed the research, H.İ.Y., E.A., İ.G.Y.K., M.B. analyzed the data. H.İ.Y., E.A. wrote the paper.

## Funding

The authors did not receive any specific funding for this study.

## Ethics Statement

Eskişehir Osmangazi University, Local Ethics Commitie approved the study protocol (Decide no: 2022/47).

## Consent

All participants gave informed consent prior to the present study.

## Conflicts of Interest

The authors declare no conflicts of interest.

## Data Availability

The data that support the findings of this study are available on request from the corresponding author. The data are not publicly available due to privacy or ethical restrictions.
